# Deception in Research on the Placebo Effect

**DOI:** 10.1371/journal.pmed.0020262

**Published:** 2005-09-06

**Authors:** Franklin G Miller, David Wendler, Leora C Swartzman

## Abstract

A common feature of research investigating the placebo effect is deception of research participants about the nature of the research. Miller and colleagues examine the ethical issues surrounding such deception.

The placebo effect is a fascinating yet puzzling phenomenon, which has challenged investigators over the past 50 years. Recently, it has been defined as the “positive physiological or psychological changes associated with the use of inert medications, sham procedures, or therapeutic symbols within a healthcare encounter” [1]. Increasing scientific inquiry has been aimed at elucidating the mechanisms responsible for placebo effects and determining how inert interventions can lead to positive changes in patients [1,2]. The majority of placebo mechanism research has been done within the context of experimental and clinical pain.

Patients' expectations for improvement, also referred to as “response expectancies,” are thought to be one of the central mechanisms responsible for placebo effects [3–5]. Brain imaging techniques are being used to explore both the neurophysiological correlates of these expectations and the mechanisms underlying placebo effects in a variety of contexts, including pain relief in healthy participants, relief of symptoms of depression, and motor functioning in patients with Parkinson disease [6–8]. Understanding these mechanisms is an important step in harnessing the placebo effect in patient care. In the words of a National Institutes of Health request for applications, “understanding how to enhance the therapeutic benefits of placebo effect in clinical practice has the potential to significantly improve healthcare” [9]. Toward that end, the National Institutes of Health invited submissions for systematic studies aimed at discerning the psychosocial factors (including expectancy) in the patient–clinician relationship and/or in the health-care environment that can potentiate healing.

A common feature of research investigating the placebo effect is deception of research participants about the nature of the research. This use of deception is considered necessary to understanding the placebo effect, but has received little systematic ethical attention. In this article, we examine ethical issues relating to deception in research on the placebo effect, with a particular focus on experiments involving patients in clinical settings. We propose a method of informing participants about the use of deception that can reconcile the scientific need for deceptive research designs with the ethical requirements for clinical research.

## Altering Expectations to Examine Placebo Mechanisms

Response expectancy is seen to be a major driving force behind the placebo effect. Therefore, a common (and some would argue, necessary) feature of research aimed at elucidating placebo mechanisms is the use of deception in experimental manipulation of participants' expectations (e.g., about whether or not they will receive a “powerful pain killer” or a “sugar pill”), while holding constant the pharmacological (or other) properties of the administered intervention. This research has clearly shown (across a wide range of clinical conditions) that altering expectancies for improvement has an impact on therapeutic outcomes [8,10–13].

The tension between scientific methods for elucidating the placebo effect and ethical norms for conducting research involving human participants is illustrated most clearly by “the balanced placebo design,” an approach designed to disentangle the relative effects of pharmacology and response expectancy. Table 1 displays the balanced placebo design in a way that highlights the deception of participants that occurs in two of the four arms of the design.[Fig pmed-0020262-g001]

**Figure pmed-0020262-g001:**
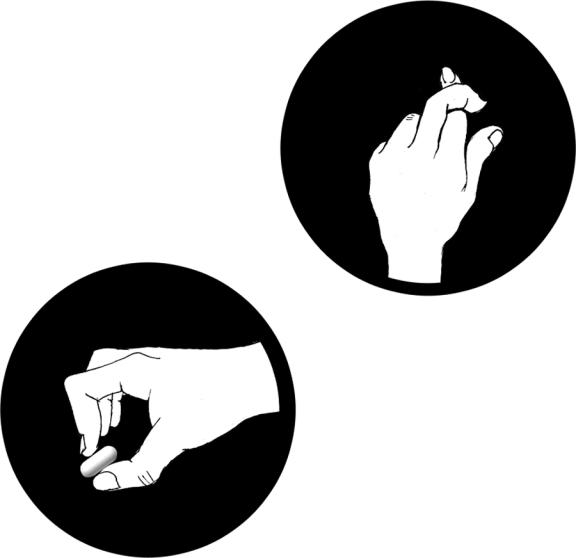
Deception of research participants is considered necessary to understanding the placebo effect—but has received little ethical attention (Illustration: Margaret Shear, Public Library of Science)

**Table 1:**
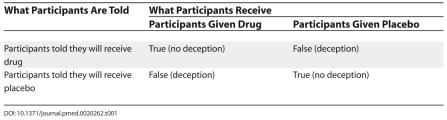
The Balanced Placebo Design

In the balanced placebo design, investigators manipulate both expectancies (e.g., informing participants that they will receive a drug versus informing them that they will receive placebo) and the pharmacological agent (giving a drug versus giving a placebo). As reviewed by Swartzman and Burkell, researchers using this paradigm with healthy volunteers have shown that expectation plays a role in the subjective and behavioral effects of a range of psychoactive substances [14]. These substances include dexamfetamine, alcohol, caffeine, nicotine, and tetrahydrocannabinol [15–19].

The balanced placebo design offers a powerful and elegant approach to evaluate drug versus expectancy effects and their interactions. As Kirsch notes, this design yields information that cannot be obtained from conventional clinical trials [20]. It provides a direct assessment of the drug effect, independent of expectancy, and the nondeceptive arms are more ecologically valid than the double-blind administration in conventional randomized trials (i.e., they mimic what goes on in the real world of clinical practice). Thus, it is not surprising that Caspi recently suggested that the balanced placebo design “be used more often in clinical trials of drug efficacy” [21]. Despite the methodological virtues of the balanced placebo design, and its prior use in healthy volunteers, we are unaware of any trials that have employed this approach with patients. Clinical investigators likely have avoided use of the balanced placebo design out of concern for the ethical acceptability of deceiving patients.

An often cited article on the balanced placebo design characterized the deception in the following way: “Although deception is involved, it is no greater than is involved in any study using placebos” [22]. However, this defense of the balanced placebo design confuses the ethical issues it raises. Placebo-controlled trials aimed at evaluating the efficacy of treatments may be regarded as having an element of deception, since the placebo control is designed to appear indistinguishable from the active treatment under investigation. Nevertheless, when these studies are conducted under effective double-blind conditions, participants are told that they will receive either a drug or a placebo, and neither the investigators nor the research participants know which intervention is received by any of the participants. Accordingly, administering the study interventions, unlike the situation of the balanced placebo design, does not involve intentionally false communication; it requires investigators to withhold information, but not to lie to participants about the interventions they will receive.

When deception is used, a conflict between the means and ends of scientific investigation ensues.

Research designed to understand the placebo effect by deceptively manipulating the expectations of participants holds great promise for understanding the psychological and neurobiological dimensions of healing. However, to pursue this research while respecting participants, it is necessary to develop an approach that reconciles the outright deception involved in placebo research, including the balanced placebo design, with the ethical norms governing clinical research.

## What Makes Deception in Scientific Investigation Ethically Problematic?

At the outset, it is useful to appreciate the conflict between the ethos of science and the use of deceptive techniques. Science aims to discover and communicate the truth about the natural world and human conduct. There are sound methodological reasons for using deception to probe for the truth about human attitudes and beliefs and their effects on behavior. It follows, however, that when deception is used, a conflict between the means and ends of scientific investigation ensues: the end of discovering the truth is pursued by the means of deliberate untruth.

It might be argued that deception in scientific investigation is no more problematic than the pervasive and accepted use of deception in daily life and in social contexts [23]. In a recent news article reporting advances in the design of computers to simulate human responsiveness, Clifford Nass, a professor of communication at Stanford University, endorses the deception involved in this project: “We spend enormous amounts of time teaching children to deceive—it's called being polite or social. The history of all advertising is about deceiving. In education, it's often important to deceive people—sometimes you say, ‘Boy you are really doing good,’ not because you meant it but because you thought it would be helpful” [24].

Deception in ordinary life typically is justified on the grounds that it is for the benefit of the individual who is being deceived. For instance, the polite and social deception that Nass cites is justified on the grounds that it is better to deceive someone slightly than to criticize the person or to hurt the person's feelings. Notice, however, that this condition is not relevant to placebo research, including the balanced placebo design. In placebo research, participants are not deceived for their own benefit. Rather, they are deceived for the benefit of science and society in general, through the development of generalizable knowledge.

Deception of research participants also clearly conflicts with the ethical norms governing clinical research [25,26]. First, it violates the principle of respect for persons by infringing on the right of prospective research participants to choose whether to participate in research based on full disclosure of relevant information. Second, it may manipulate individuals to volunteer when they otherwise would not have chosen to do so had they been informed accurately about the nature of the research, including its use of deception. For these reasons, deception, as it is currently practiced in the conduct of research on the placebo effect, is incompatible with informed consent.

Third, although scant systematic data have been collected on the effects of deception on clinical research participants, some available evidence indicates that when the deception is revealed, as in the debriefing process that often accompanies deceptive research, it causes distress to at least some participants [27]. The adverse impact of deception in psychological research, and whether it can be reversed adequately through a debriefing process, is a subject of debate [28–31]. Furthermore, deception in research involving patients in clinical settings may prove more upsetting. This is because participants in deceptive psychological research are, for the most part, psychology undergraduates who often are aware that deception is sometimes used in psychological research [32]. Patients, in contrast, legitimately expect to be able to trust in, and receive truthful communication from, clinicians and clinical investigators. This trust is violated by the use of deception. Especially problematic is the use of deception in experiments conducted by clinicians who have a prior clinician–patient relationship with the patients enrolled in the study. When patients learn about the use of deception in the process of debriefing, which is a common feature of deception research, they may feel that their trust has been violated. Consequently, deception of patients may have deleterious effects on the willingness of patients to volunteer for future clinical research. More importantly, by undermining patients' faith in the truthfulness of physicians, deception might interfere with the future medical care of those who have experienced deceptive research.

Deception may be harmful not only to those who are deceived but also to those who practice it.

Finally, deception in research raises ethical concern because it can be corrupting for the professionals who practice it, and for those who witness it. According to an ancient perspective in moral philosophy, moral character depends on habits of conduct [33]. The use of deception in research may interfere with the disposition not to lie or deceive persons. This problem is compounded when the study design requires deception at the initiation of the trial as well as repeated deception of participants while conducting the research. Those who witness deception, especially if performed or sanctioned by professionals in positions of authority, may develop skewed perceptions of the ethics of deception, which may have negative consequences for the development of moral character. In sum, deception in research is prima facie wrongful, and it may be harmful not only to those who are deceived but also to those who practice or witness it.

The American Psychological Association's guidelines [34] are perhaps the most prominent attempt to reconcile the use of deception with the ethical norms of human participant research. According to guideline 8.07 (Deception in Research), “(a) psychologists do not conduct a study involving deception unless they have determined that the use of deceptive techniques is justified by the study's significant prospective scientific, educational, or applied value and that effective nondeceptive alternative procedures are not feasible; (b) psychologists do not deceive prospective participants about research that is reasonably expected to cause physical pain or severe emotional distress; (c) psychologists explain any deception that is an integral feature of the design and conduct of an experiment to participants as early as is feasible, preferably at the conclusion of their participation, but no later than at the conclusion of the data collection, and permit participants to withdraw their data.”

We have argued elsewhere that these three conditions are not sufficient to address the ethical concerns raised by deceptive research [25,26]. In particular, these conditions fail to address the fact that concealing the use of deception itself may affect individuals' decision to participate in research and precludes individuals from deciding whether they want to participate in deceptive research. To be sure, the use of debriefing may mitigate the potential harmful consequences of deceitful communication by explaining the rationale for deception. However, just as compensation for damages caused by negligence or restitution for crime does not cancel an infringement of a person's rights, debriefing does not cancel the violation of the principle of respect for persons. To consider how these ethical concerns arise in actual practice, and what steps might be taken to address them, it will be helpful to consider specific examples of the use of deception in placebo research (Table S1).

## Examples of Deception in Placebo Research

First, in an experiment investigating suggestion and expectation relating to placebo analgesia, 13 women with irritable bowel syndrome were recruited, and were subjected to visceral pain evoked by rectal distention, using a balloon attached to a rectal catheter. The experiment took place under five experimental conditions: (1) natural history (no intervention relating to or disclosure about the pain stimulus), (2) rectal placebo (a sterile surgical lubricant placed on the balloon, described as effective in relieving pain), (3) rectal lidocaine, (4) oral lidocaine, and (5) rectal nocebo (a placebo intervention accompanied by a disclosure that the intervention often causes increased pain) [13]. Notably, the research report stated that “the gastroenterologist who performed the study was the doctor the patients normally consulted in the clinic.”

The investigators described their disclosure to the participants as follows: “The patients were told that four drugs that reduced and increased pain in relation to IBS [irritable bowel syndrome], respectively, were being tested, and that they had been proven effective in preliminary studies” [13]. In reality, the participants were administered two different forms of only one drug, along with two placebos. Hence, the participants were deceived by being informed that they would receive drugs that in fact were placebo interventions.

Second, investigators recruited patients with asthma, from an academic medical center, to participate in an experiment examining changes in forced expiratory volume in one second following administration of inhaled saline described deceptively as either a bronchoconstrictor or a bronchodilator [12]. The purpose was to determine the impact of suggestion on a placebo intervention in patients identified as suggestible or suggestion-resistant, based on a validated rating scale. The disclosure to the research participants was described in the article reporting the experimental results as follows: “Patients were contacted via telephone and informed that the investigators were hoping to understand how medications produce beneficial effects in asthma, including whether telling subjects about the potential effects of various medications would alter response to these agents. Patients were not told that they would be exposed to placebo interventions.” The study thus used elaborate deception, which involved an inaccurate account of the nature of the research and false descriptions of a placebo intervention. It is therefore puzzling that the authors reported that “all patients gave informed consent to participate in the study,” especially since there was no indication that the participants were informed that deception would be employed. Instead, the participants were debriefed about the study at the end of the experiment.

## Authorized Deception

Can deceptive research be made compatible with informed consent? Use of deception is not consistent with fully informed consent. If participants are told the true purpose of research and the nature of all procedures, there would be no deception. However, participants can be informed prior to deciding whether to volunteer for a study that the experimental procedures will not be described accurately or that some features of these procedures will or may be misleading or deceptive [25,26]. This approach, which we call “authorized deception,” permits research participants to decide whether they wish to participate in research involving deception and, if so, to knowingly authorize its use. Authorized deception is compatible with the spirit of informed consent. It fosters respect for persons, despite the use of deception, by alerting prospective participants to the fact that some or all participants will be deliberately deceived about the purpose of the research or the nature of research procedures.

For example, investigators using the balanced placebo design to study expectancy and pharmacological effects of dexamfetamine described the informed consent disclosure as follows: “For ethical reasons it was stated in the consent form that ‘…some information and/or instructions given [to the participant] may be inaccurate’” [15]. This statement recognizes the ethical force of authorized deception, but does not seem to go far enough. As illustrated above, the balanced placebo design involves lying to participants in two arms of the study: some participants are told that they are being administered a particular drug when in fact they receive placebo, and others that they are being administered placebo when in fact they receive the drug. Consequently, it is at best an understatement to describe the disclosure in this experiment as possibly involving “inaccurate” information. It would be more accurate to inform the prospective participants that some research participants will be misled or deceived.

Use of deception is not consistent with fully informed consent.

Variants of the authorized deception approach have been advocated, and sometimes evaluated experimentally, since the 1970s [23,35–37]; however, it has not become a routine feature of research using deception [38]. In order to solicit informed authorization for the use of deception, the informed consent document could be worded as follows: “You should be aware that the investigators have intentionally misdescribed certain aspects of the study. This use of deception is necessary to obtain valid results. However, an independent ethics committee has determined that this consent form accurately describes the major risks and benefits of the study. The investigator will explain the misdescribed aspects of the study to you at the end of your participation.”

When deception of study participants is necessary and justified by the scientific value of the study, the use of authorized deception makes the process of deceptive research transparent. Participants are informed that they will be misled or deceived, though obviously the exact nature of the deception cannot be disclosed. They are assured that the research has been reviewed and approved by an ethics oversight committee that has no vested interest in the research in question, and that no important risks, other than the risks of the deception itself, have been concealed. Finally, they are informed that debriefing will occur.

## Methodological Objections and the Need for Future Study

One possible objection to the technique of authorized deception is that it is liable to defeat the purpose of using deception to obviate potentially biased responses of research participants to research interventions. Informing participants that deception will occur (particularly in a study that involves administration of a placebo) is apt to make them suspicious and wary, thus possibly contributing to biased data. This methodological risk is avoided in most deceptive research, which does not employ this technique, provided that prospective participants do not otherwise suspect that deception will be used. However, limited available research indicates that the anticipated biased results from disclosing the possibility of deception do not necessarily occur.

Holmes and Bennett assessed this methodological concern experimentally. Psychology students were exposed to a deceptive experiment in which they were falsely informed that two to eight “painful electric shocks” would be administered at random times after a red signal light appeared [35]. No shocks actually were administered. Measures of self-reported anxiety and physiological arousal (pulse and respiration rates) were obtained. Prior to the deceptive shock intervention, one experimental group was informed that deception is occasionally used in psychology experiments to assure unbiased responses. The other group exposed to the deceptive shock intervention did not receive any information about the possibility of deception. No outcome differences were observed for participants informed of the possibility of deception versus those not informed.

The information about deception in this experiment, however, falls short of the authorized deception approach that we recommend. It disclosed to prospective participants that deception is a possibility in “a few experiments,” rather than informing them that deception would actually be employed for all or some participants in the particular experiment in which they were invited to enroll. In contrast, Wiener and Erker directly tested the authorized deception approach, described as “prebriefing,” in an experiment evaluating attributions of responsibility for rape based on transcripts from an actual rape trial [37]. Participants (68 undergraduate psychology students) were either correctly informed or misinformed about the jury verdict regarding the defendant's guilt. Half of participants received an informed-consent document stating that “you may be purposefully misinformed.” The other half was not alerted to the possibility of deception. No differences on attribution of responsibility were observed depending on whether or not the participants were prebriefed about the use of deception.

The effects of the authorized deception approach on study outcomes merit investigation.

A second methodological objection to authorized deception is that it has the potential to reduce the comparability to previous research on placebo mechanisms that did not employ this technique. There is no way to avoid this problem. But to argue that consequently the authorized deception approach should not be adopted would suggest that past ethical lapses justify current ethically deficient practice. Finally, disclosure of the use of deception may lead to reduced participant enrollment, making it more difficult to complete valuable studies and possibly reducing their generalizability. At the extreme, it is possible that too few prospective participants will be willing to volunteer, especially for experiments recruiting patients. One clinical research study using the authorized deception approach (in this case, informing participants that details about the purpose of the research were withheld) found no substantial impact on enrollment [39]. This remains to be studied further. But if this approach reduces participant enrollment, it would indicate that eligible prospective participants do not wish to be deceived, casting doubts on the legitimacy of using deception without disclosing its use.

The results of psychology experiments that alerted participants to the possibility of deception and used prebriefing are encouraging, but may not be generalizable to the situation of patients in clinical research. The null findings obtained by Weiner and Erker and Holmes and Bennet need to be interpreted with caution [35,37]. Given that their study participants were psychology undergraduates, even those who were not prebriefed about the use of deception could have anticipated that they might be deceived [32].

Accordingly, the effects of the authorized deception approach on study outcomes merit investigation with respect to research on the placebo effect in a clinical setting. For example, a methodological experiment comparing the authorized deception approach to the traditional approach that does not reveal the use of deception might be attached to a study using the balanced placebo design to evaluate expectation effects relating to placebo analgesia among patients recovering from surgery. Patients would be randomized to the two methods of disclosure, which would be assessed in terms of their impact on reported pain relief among patients in the various arms of the underlying study. This would allow investigators to examine the extent to which the authorized deception approach biases the study outcomes. It might be desirable to conduct such a methodological experiment in connection with a diversity of underlying studies of the placebo effect and in various patient populations.

We suspect that the use of authorized deception will not bias studies of the placebo effect. Hence, the results of such experiments have the potential to pave the way for important research to proceed that uses the balanced placebo design in the clinical setting along with the authorized deception approach—research that otherwise might be rejected by ethics review committees, owing to concerns about using deception in clinical research. If authorized deception does produce some bias, decisions will have to be made by investigators and ethics review committees about the importance of this bias in compromising the validity of the research compared to the importance of respecting the autonomy of research participants. Conducting studies to estimate the extent of the bias will facilitate and inform these decisions.

If the use of authorized deception proved to produce seriously biased results, then it might be argued that it would be unethical to use the balanced placebo design in clinical research, owing to the extensive deception involved. Nevertheless, some aspects of the role of expectancy in therapeutic responses could still be evaluated in an ethical manner by using nondeceptive research paradigms in clinical settings [20,40,41], such as comparing an open versus closed [10,42] or an open versus double-blind administration of a therapeutic agent [11]. The problem with these experimental paradigms, however, is that because they do not fully manipulate expectancy and pharmacology in a factorial design (as does the balanced placebo design), they do not permit a rigorous evaluation of drug versus expectancy effects and their interaction.

## Remaining Qualms about Deceptive Research

Deceptive research involving patients in the clinical setting might be considered unethical even when all pertinent safeguards are in place, including the use of authorized deception. This is because deception, even if authorized in advance, violates the ethical framework of the clinician–patient relationship, which is based on trust. It may be argued that clinician investigators who deceive patients in the course of research are acting fraudulently. Accordingly, professional ethics precludes participation in deceptive research.

This objection, however, confuses the ethics of clinical research with the ethics of medical care [43,44]. Clinical research aims at developing generalizable knowledge in order to improve medical care in the future. Promoting the medical best interests of particular patients is not part of the primary purpose of clinical research. Clinical research also departs from the ethics of medical care in the methods it uses, such as randomization, double-blind procedures, placebo controls, and the justification of risks. Nearly all clinical research, especially research that is not aimed at evaluating the efficacy or safety of treatment interventions, poses risks to participants that are not offset by potential medical benefits. Accordingly, the researcher is not functioning as a therapist in the context of clinical research. It follows that deceptive behavior that would be fraudulent in clinical practice is not necessarily unethical in clinical research. The informed-consent process should clarify that the research in question is different from and outside the purview of medical care. The use of authorized deception in this context makes research involving deception consistent with ethical guidelines appropriate to clinical research.

Experiments investigating the placebo effect evoke legitimate ethical concerns.

This objection cannot be so readily dismissed, however, if the investigator or members of the team of investigators include clinicians who have a prior therapeutic relationship with research participants, as in the experiment described earlier involving patients with irritable bowel syndrome [13]. When investigators simultaneously have both therapeutic and research roles, it is difficult, if not impossible, to avoid the violation of medical ethics constituted by deception, even if adequate safeguards are in place to make the deception justifiable in the context of research. In addition, the potential for negative consequences to patients from deception is likely to be greater in this situation. It is not clear why it would be necessary for a clinician having a prior therapeutic relationship with participants to conduct valuable research on the placebo effect. For example, in the case of Vase et al.'s irritable bowel syndrome experiment, an experienced clinician would be needed to safely administer the rectal distention procedure; however, someone other than the treating physician could be recruited to perform this function.

## Conclusion

Research aimed at elucidating the placebo effect promises to produce valuable knowledge concerning the psychological and neurobiological dimensions of healing. Insights gleaned from this research may contribute to the development of clinical interventions that can enhance the therapeutic efficacy of existing treatments. Experiments investigating the placebo effect, however, evoke legitimate ethical concerns, owing to the use of deception.

Key safeguards to assure the ethical design and conduct of deceptive placebo research include (1) prior review and approval by an independent research ethics committee to determine that the use of deception is methodologically necessary and that the study protocol offers sufficient value to justify the risks it poses to participants, including the use of deception; (2) disclosure in the informed-consent document that the study involves the use of deception; and (3) debriefing of participants at the conclusion of research participation. To contribute to public accountability, articles reporting the results of research using deception should describe briefly adherence with these participant-protection guidelines [45,46]. As in all clinical research, an acceptable balance must be struck between promoting valuable knowledge and protecting the rights and well-being of participants.

## Supporting Information

Table S1 Clinical Studies on the Placebo Effect Involving Deception (69 KB DOC).
