# Supplementary material for: Deception in Research on the Placebo Effect
Source: PLoS Med. 2005 Sep 6;2(9):e262. doi: 10.1371/journal.pmed.0020262 (PMC1198039; doi:10.1371/journal.pmed.0020262)
Supplement: Supplementary file 1 [file pmed.0020262.st001.doc]

# **Table S1.** Clinical Studies on the Placebo Effect Involving Deception

| Study and Sample | Aim of Study | Study Design | Nature of Deception | Consent/ Debriefing |
| --- | --- | --- | --- | --- |
| Lee et al. [47]  Individuals with coughs associated with acute upper respiratory tract infection. Recruited from staff and students of a university and from the general population; N=54. | To determine “the effects of a placebo treatment on cough in patients with cough associated with acute upper respiratory tract infection.” | Double- or single-blind RCT; vitamin E (3000 IU) gelatin capsule (served as the placebo), taken with water vs. water only. Duration: A 77 minute session, comprised of three subsessions: (1) pre-treatment cough recording (31 minutes), (2) treatment (15 minutes), (3) post-treatment cough recording (31 minutes). | Participants were told that the aim of the study was to investigate the effects of vitamin E treatment on cough. However, the real goal of the study was to examine the effect of placebo on cough, and vitamin E was given as a placebo and not as an active treatment. | Informed consent: “All patients gave written informed consent for the study.”  Authorized deception: No  Debriefing: No mention |
| Benedetti et al. [11]  Individuals with Parkinson disease, who had electrodes surgically implanted bilaterally in their subthalamic nuclei; N=10. | To examine the roles of “conscious expectation and unconscious conditioning in motor placebo/ nocebo responses.” | Double-blind crossover. Three experimental conditions on three different days, in varied orders: group 1: stimulator on, no instructions; group 2: stimulator on, patient told it was turned off (nocebo); group 3: stimulator on, with intensity unchanged, patient told intensity was going to increase, thereby, improving effectiveness (placebo). | Misinformed: Group 2 was told that stimulator was being turned off, whereas it actually was left on. Group 3 was told that stimulator intensity was increased, whereas it actually remained the same. | Informed consent: “Written informed consent was obtained.”  Authorized deception: No  Debriefing: No mention |
| Leigh et al. [12]  Asthma patients; N=17. | To examine “whether suggestion differentially influenced airway tone in a group of highly suggestible asthmatics.” | Double-blind crossover; suggestible (n=8) vs. suggestion-resistant (n=9) participants. Baseline FEV1, followed by three bronchial response tests every 45 minutes: test 1: given saline, told bronchoconstrictor; test 2: given saline, told new bronchodilator; test 3: given methacholine, “without any suggestion.” | Information withheld: “Participants were not told that they would be exposed to placebo interventions.”  Misinformed: Test 1: Were given saline but were told “inhaled solution might make their airway become tighter and cause them to wheeze.”  Test 2: Were given saline but were told “Inhaling the drug should cause their airways to become less tight & allow their breathing to feel much easier”. | Informed consent: “All patients gave informed consent to participate”  Authorized deception: No  Debriefing: “…received individual debriefing at the end of the visit.” |
| Vase et al. [13]  Women with irritable bowel syndrome; N=13. | To examine the extent to which “verbal placebo/ nocebo suggestions influence evoked rectal pain and evoked cutaneous heat pain in IBS patients.” | Crossover design; five sessions, five days apart: natural history, rectal placebo, rectal nocebo, rectal lidocaine, oral lidocaine. | Misinformed: Rectal Placebo group told: “agent you have just been given is known to significantly reduce pain in some patients.”. Rectal Nocebo group told: “the agent you have just been given is known to significantly increase pain in some patients.” | Informed consent: “All patients signed informed consents.”  Authorized deception: No  Debriefing: “…were debriefed after sessions were complete.” |
| De Craen et al. [48]  Patients attending a chronic pain outpatient clinic; N=112 . | To examine whether “experimentally induced expectancy can modify the effect of tramadol relative to placebo.” | Double-blind RCT; single-dose tramadol vs. placebo, given with positive vs. neutral instructions. | Misinformed: Placebo with positive instructions: “…this drug is very effective and will decrease the pain quickly after taking it.”  Tramadol with neutral instructions: “It will not be beneficial in all patients.” | Informed consent: “..informed consent [was] obtained..”  Authorized deception: “…it was stated that there was a second objective of this research, and that it was important that the patients were not aware of the content of this objective.”  Debriefing: No mention |
| Pollo et al. [4]  Lung cancer patients undergoing posterolateral thoracotomy; N=38. | To examine “...the role of response expectancies in the clinical pharmacological setting.” | RCT; group 1: given saline, told it was “rehydrating solution.”; group 2: given either saline or buprenorphine, told it could be either a painkiller or placebo. (classic double-blind instructions); group 3: given either saline or buprenorphine, told it was a painkiller. (deceptive administration). | Misinformed: Half of those in group 3 were given saline, but were told they were getting a “powerful painkiller.” | Informed consent: “Patients..gave their informed consent to receive either a painkiller or a placebo.”  Authorized deception: No  Debriefing: No mention |
| Goodenough et al. [49]  Children (aged 3–17) undergoing venipuncture; N=117. | To examine “the role of placebo cream in reducing needle-related pain.” | RCT (single or double blind); no cream vs. inert cream vs. inert cream and suggestion. | Misinformed: Inert cream and suggestion group told: “We are trying out a new special cream. I am going to put some cream on your arm that might make it [the needle] hurt less.” | Informed consent: “Children were approached and agreed to participate.”  Authorized deception: No  Debriefing: No mention |
| Benedetti et al. [50]  Patients undergoing posterolateral thoracotomy for lung surgery; N=93. | To examine “the effect of proglumide (a CCK antagonist) on the placebo response in postoperative pain.” | Double- or single-blind RCT; infusion of proglumide vs. saline; open versus hidden; duration: one hour (two hours post-surgery). | Misinformed:  In the open conditions for proglumide (n=27) and placebo (n=13), participants were told they were getting a “powerful painkiller.” | Informed consent:  “Informed consent was obtained.”  Authorized deception: No  Debriefing: No mention |
| Bergmann et al. [51]  Hospitalized cancer patients experiencing mild or moderate pain not requiring opiates; N=49 | To examine “whether informed consent in a therapeutic trial modifies the analgesic effect of naproxen and placebo.” | Crossover double- blind; placebo vs. naproxen; and informed vs. uninformed; duration: three hours on two consecutive days. | Information withheld:  Half of the participants did not know they were participating in a clinical trial. | Informed consent:  Half of the participants did not consent to being in a clinical trial.  Authorized deception: No  Debriefing: No mention |
| Hashish et al.[52]  Dental patients undergoing third lower molar extraction; N=125 | To examine “...mechanisms by which ultrasound (US) treatment reduces pain.” | Single- or double- blind RCT.  1. US & massage  2. Mock US & massage  3. Mock US  4. Self-massage  5. No treatment control | Misinformed:  Groups 2 and 3 got mock US, but were told that “…they were to receive ultrasound which would help reduce pain and swelling and they need take no active part.” | Informed consent:  “Patients participating…did so with their informed consent.”  Authorized deception: No  Debriefing: No mention |
| Butler and Steptoe [53]  Asthmatics; N=12 | To examine “the effects of placebo medication on pulmonary responses in asthmatics,…and... the roles of expectancy and anxiety.” | Single-blind crossover.  Three weekly sessions, with 2nd and 3rd sessions counter-balanced. In all sessions, participants inhaled only nebulized water (H_2_O), but were told the following: session 1: inhaling H_2_O; session 2: inhaling bronchoconstrictor, pre-treated with drug; session 3: inhaling bronchoconstrictor, pre-treated with a nonactive substance. | Misinformed:  In sessions 2 and 3, participants inhaled nebulized water, but were told it was a bronchoconstrictor.  In session 2, participants inhaled nebulized water prior to getting the ‘bronchoconstrictor’, but were told this water was a powerful new drug. | Informed consent: No mention  Authorized deception: No  Debriefing: No mention |
| Luparello et al. [54]  Asthmatics; N=20 | “To better delineate the influences of expectation on the airway response of asthmatics to the inhalation of broncho-active pharmaco-logical agents.” | Double-blind crossover; four testing sessions, in randomized order, each at least one day apart; given bronchoconstrictor vs. bronchodilator, and told it was bronchoconstrictor vs. bronchodilator. | Misinformed: Given bronchoconstrictor and told: “This is a broncho-dilator…that will ...make it easier for you to breathe.” Given bronchodilator and told: “This is a broncho-constrictor… that will ….make it harder for you to breathe.” | Informed consent: No mention  Authorized deception: No  Debriefing: No mention |

RCT, randomized controlled trial; US, ultrasound.

**References**

Reference numbers refer to the references in the associated paper, plus the following additional references.

47. Lee PCL, Jawad MSM, Hull JD, West WH, Shaw K, et al. (2005) The antitussive effect of placebo treatment on cough associated with acute upper respiratory infection. Psychosom Med 67: 314–317.

48. de Craen AJ, Lampe-Schoenmaeckers AJ, Kraal JW, Tijssen JG, Kleijnen J (2001) Impact of experimentally-induced expectancy on the analgesic efficacy of tramadol in chronic pain patients: A 2 × 2 factorial, randomized, placebo-controlled, double-blind trial. J Pain Symptom Manage 21: 210–217.

49. Goodenough B, Kampel L, Champion GD, Nicholas MK, Ziegler JB, et al. (1997) An investigation of the placebo effect and age-related factors in the report of needle pain from venipuncture in children. Pain 72: 383–391.

50. Benedetti F, Amanzio M, Maggi G (1995) Potentiation of placebo analgesia by proglumide. Lancet 346: 1231.

51. Bergmann JF, Chassany O, Gandiol J, Deblois P, Kanis JA, et al. (1994) A randomized clinical-trial of the effect of informed consent on the analgesic activity of placebo. Clin Trials Metaanal 29: 41–47.

52. Hashish A, Hai HK, Harvey W, Feinmann C, Harris M (1988) Reduction of post-operative pain and swelling by ultrasound treatment: A placebo effect. Pain 33: 303–311.

53. Butler C, Steptoe A (1986) Placebo responses: An experimental study of psychophysiological processes in asthmatic children. Br J Clin Psychol 25: 173–183.

54. Luparello TJ, Leist N, Louise CH, Sweet P (1970) The interaction of psychologic stimuli and pharmacological agents on airway reactivity in asthmatic subjects. Psychosom Med 32: 509–513.
